# Accelerations Recorded by Simple Inertial Measurement Units with Low Sampling Frequency Can Differentiate between Individuals with and without Knee Osteoarthritis: Implications for Remote Health Care

**DOI:** 10.3390/s23052734

**Published:** 2023-03-02

**Authors:** Arash Ghaffari, John Rasmussen, Søren Kold, Rikke Emilie Kildahl Lauritsen, Andreas Kappel, Ole Rahbek

**Affiliations:** 1 Interdisciplinary Orthopaedics, Aalborg University Hospital, 9000 Aalborg, Denmark; 2 Department of Materials and Production, Aalborg University, 9220 Aalborg East, Denmark

**Keywords:** inertial measurement unit, wearable technology, telemedicine, digital health, knee osteoarthritis, gait analysis, LASSO regression

## Abstract

Determining the presence and severity of knee osteoarthritis (OA) is a valuable application of inertial measurement units (IMUs) in the remote monitoring of patients. This study aimed to employ the Fourier representation of IMU signals to differentiate between individuals with and without knee OA. We included 27 patients with unilateral knee osteoarthritis (15 females) and 18 healthy controls (11 females). Gait acceleration signals were recorded during overground walking. We obtained the frequency features of the signals using the Fourier transform. The logistic LASSO regression was employed on the frequency domain features as well as the participant’s age, sex, and BMI to distinguish between the acceleration data from individuals with and without knee OA. The model’s accuracy was estimated by 10-fold cross-validation. The frequency contents of the signals were different between the two groups. The average accuracy of the classification model using the frequency features was 0.91 ± 0.01. The distribution of the selected features in the final model differed between patients with different severity of knee OA. In this study, we demonstrated that using logistic LASSO regression on the Fourier representation of acceleration signals can accurately determine the presence of knee OA.

## 1. Introduction

In patients with knee osteoarthritis (OA), gait analysis can support clinical decisions and contribute to the evaluation of interventions by providing relevant information on the course of the disease and the response to treatment [1]. Objective gait analysis has traditionally been limited to sophisticated biomechanical gait laboratories, but recently, inertial measurement units (IMUs) have received more attention due to their several advantages, especially for use in natural everyday settings in people’s daily lives [2]. However, compared with conventional methods, these sensors provide restricted data, signifying the importance of comprehensive data analysis for the practical application of IMUs in clinical settings.

One of the most useful clinical applications of IMUs, especially in telemedicine and remote patient monitoring, is determining the presence or severity of knee OA. Different studies used various data analysis methods for this purpose [3]. While some studies employed more computationally complex approaches to process the IMU-derived data [4,5,6,7], others primarily used raw IMU data to classify gait deviation due to knee OA [8,9,10]. The complex methods yield spatiotemporal parameters and joint kinematics comparable to traditional gait analysis; however, some concerns have been raised about the validity of the results of the complex methods, in addition to the high computational costs and the need for using multiple sensors simultaneously [3]. Analyzing raw sensor data, as an alternative method, relies on defining discrete parameters within the waveforms (such as ranges and peak values)—ignoring most of the information contained in the signal [11]—or employing various time-continuous analyses [12,13,14]. Time-continuous analyses, even though enabling waveforms’ overall shape and characteristics assessment, are sensitive to time variability and the definition of stride [11,15].

Alternatively, the Fourier representation of a signal has been suggested as a quantitative analysis of the entire gait waveform [16,17] with several advantages such as simplifying the comparison and evaluation of the signals, minimizing the possible inter- and intra-experimental errors, and facilitating efficient data storage and reconstruction of the original signal [18,19,20]. These advantages can address the challenges mentioned earlier with IMU data analysis, especially regarding patients’ remote monitoring over extended periods. However, it is still unclear whether sensors suitable for long-term data recording (i.e., sensors with longer battery life and, therefore, lower sampling rate and inferior accuracy) can detect and differentiate walking disorders. In addition, the significance of the Fourier coefficients in various gait conditions, such as knee OA, has not been entirely investigated, while its clarification would enable the use of noninvasive IMUs as simple and inexpensive tools for long-term remote monitoring of patients.

Therefore, the primary objective of this study was to determine whether simple and low-sampling frequency IMUs designed for long-term data collection can differentiate between individuals with and without knee OA. We investigated the features from the frequency domain representation of the lower limbs’ linear acceleration signals. In addition, we aimed to identify the frequency domain features most related to the disease and compare those features in patients with different severity of knee OA.

## 2. Materials and Methods

### 2.1. Study Setting and Participants

This cross-sectional observational study was conducted at Aalborg University Hospital, Denmark. Data collection was performed at the hospital’s outpatient clinic. The participants included 27 patients with unilateral knee osteoarthritis and 18 volunteers without lower limb complaints. The exclusion criteria were a BMI higher than 35 kg/m^2^, a recent history of surgery in the lower limbs, neurological movement disorders, and inflammatory arthritis. In addition, we excluded the patients with complaints of pain or discomfort in the spine and lower-limb joints other than the affected knee and healthy controls with any pain or discomfort in the spine or lower-limb joints. Orthopedic surgeons with a subspecialty in knee replacement surgery established the diagnosis of knee OA in the patients. The Regional Committee on Health Research Ethics approved the study (journal 2021-000438). All participants were informed about the study and signed informed consent forms.

### 2.2. Data Collection

The participants’ basic information (age, sex, and BMI) was registered in a secure REDCap database hosted by North Jutland Region. The participants also filled out the knee injury and osteoarthritis outcome score (KOOS) questionnaire as a subjective measure of the problems regarding knee OA [21]. KOOS scores were analyzed separately in five subscales: pain, symptoms (other than pain), disability regarding the activities of daily living (ADL), disability regarding sport and recreational activities (more demanding than activities of daily living), and quality of life (QoL). In addition, the severity of knee OA in the patients’ radiographic images was evaluated according to the Kellgren–Lawrence (KL) classification [22].

**Figure 1:**
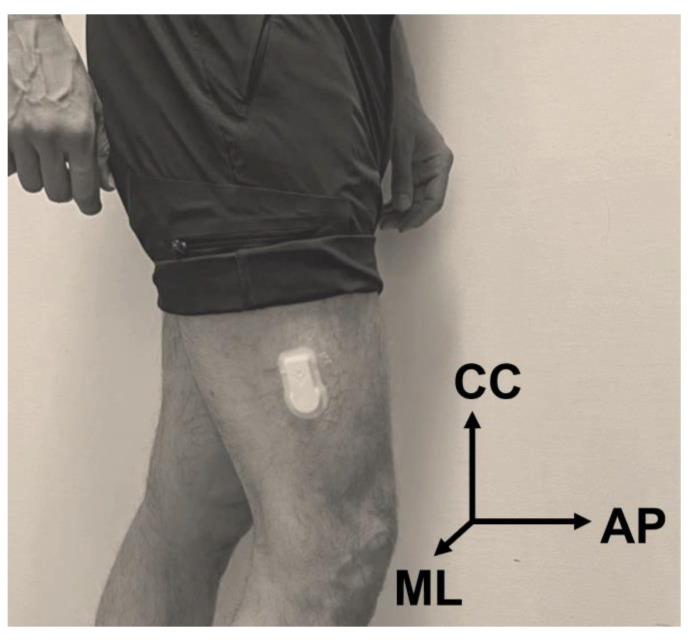
The photograph demonstrates the employed inertial measurement unit (IMU) at its location on the lateral distal side of the thigh and the corresponding coordinate axes (CC: craniocaudal, AP: anteroposterior, ML: mediolateral).

The IMUs were SENS Motion sensors (SENS Motion^®^, Copenhagen, Denmark) containing only a 3D accelerometer sampling at 12.5 Hz and were previously validated [23,24]. We placed the IMUs on the lateral side of the distal thigh, ipsilateral with the affected knee (Figure 1). According to the manufacturer’s instructions, the sensors were located approximately 10 cm above the lateral femoral epicondyle, and no calibrations were performed before recording data. The side of the IMU in the control group was randomly chosen. The participants performed two overground walking trials at a self-selected speed with a 5 min interval in a straight corridor.

### 2.3. Acceleration Signal Processing

Three-dimensional linear acceleration signals from the IMUs corresponding to craniocaudal (CC), anteroposterior (AP), and mediolateral (ML) axes were recorded and processed for further analysis. We randomly selected each participant’s first or second gait trial to analyze the data. Considering the periodic nature of gait kinematic signals, we reconstructed a continuous interpolation of the acceleration signals using the Fourier method. Since averaged waveform is more reliable and the average variance provides additional information as to the randomness of the variable [25], we calculated the average of ten gait cycles extracted in the middle of the walking bout and segmented it into ten individual cycles using autocorrelation. Subsequently, the fundamental angular stride frequency (ω), the Fourier series representation, and the power of the signals corresponding to the signal frequencies were obtained from the averaged signal. The power of a signal at a particular frequency, *P*(*f_i_*), reveals how much of that frequency, *f_i_*, is present in the signal and calculated by adding the squares of the *i*th pairs of Fourier coefficients. The calculation of the Fourier coefficients and the power of the signal was previously described by Derrick [26].

The value of the Fourier coefficients and the power at the first six frequencies, *P*(*f*_1_), *P*(*f*_2_), *P*(*f*_3_), …, *P*(*f*_6_), were calculated for the signals corresponding to the CC, AP, and ML axes.

Numerical processing of the acceleration signals was performed in Python [27].

### 2.4. Logistic LASSO Regression

Logistic LASSO (least absolute shrinkage and selection operator) regression was employed in this study. The LASSO is a regularization method that performs classification tasks by selecting the most relevant features to the outcome variable, i.e., knee OA. This shrinkage method can actively select from a large and potentially multicollinear set of variables in the regression, resulting in a more relevant and interpretable set of predictors [28]. In addition, LASSO minimizes the regression coefficients to reduce the likelihood of overfitting, and as regression method can handle the confounders and the correlation within the gait data.

Since we did not perform any matching between the patients and the control group, the potential confounders (ω, age, sex, and BMI) were added to the regression model, in addition to the power of 18 signal frequencies along the CC, AP, and ML axes as the explanatory variables (Table 1). The outcome variable was the participant’s group (patients vs. controls) considered as the presence of knee OA.

To construct the features from the explanatory variables, the continuous variables (age, BMI, ω, and the power of the signal frequencies) were standardized by removing the mean and scaling to unit variance, and the only categorical variable (sex) was encoded as a factor with unordered levels.

**Figure 2:**
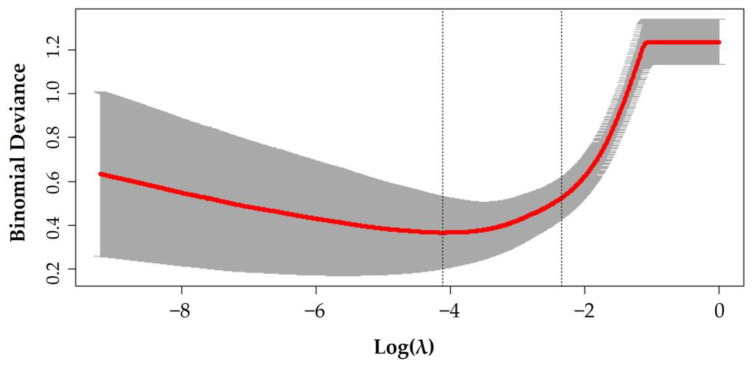
Cross-validation plot for the penalty coefficient (λ). Two vertical dashed lines correspond to the λ_min_ and λ_1se_ to the left and right, respectively. The x-axis regards the logarithm of λ.

The model was a logistic LASSO regression model fitted via penalized maximum likelihood. The penalty coefficients (λ) were computed using 10-fold cross-validation with values ranging from 10^−12^ to 10^2^, based on best computed binomial deviances. No weight or offset was specified for the observations. Two λ values were computed: λ_min_, defined as λ that minimizes the binomial deviance, and a more stringent value of λ_1se_, defined as the largest λ that is still within one standard error of the minimum binomial deviance. λ_1se_ results in a smaller number of covariates than λ_min_. We estimated λ_min_ = 0.02 and λ_1se_ = 0.10 (Figure 2).

We utilized R Statistical Software [29] and the related packages to fit the logistic LASSO regression model [30,31] and to estimate the confidence intervals for the coefficients in the model [32].

The LASSO coefficients’ 95% CI and *p*-values at a fixed value for the penalty parameter (λ = λ_1se_) were obtained using the method described by Taylor and Tibshirani [33].

Finally, the model’s performance in classifying the gait signals into osteoarthritic and non-osteoarthritic knees (patients vs. controls) was estimated by performing 10-fold cross-validation on divided data into 75% training and 25% validation sets. Subsequently, the mean and 95% CI were calculated for accuracy as the percentage of correctly classified instances out of all cases and Cohen’s Kappa as the measure of agreement between the actual and classified labels. We have also calculated the accuracy and Kappa for a separate logistic LASSO regression model with potential confounder variables (age, BMI, and ω) to ascertain the superior ability of the power of the frequencies than the potential confounder variables in classifying the gait signals.

### 2.5. Comparing the Severity of Knee OA

We used KOOS as a subjective measure to estimate the severity of individuals’ problems related to knee OA. We divided the participants into three groups using terciles (33rd and 67th percentiles) for each of the KOOS subscales (pain, symptoms, ADL, sport, and QoL): Individuals with the highest scores or no/mild knee OA (G0), individuals with scores in the middle range or moderate knee OA (G1), and individuals with lowest scores or severe knee OA (G2). We also divided the participants into three groups based on the radiographic classification of knee OA. However, since we did not perform a radiographic examination in healthy individuals, the participants in the control group, patients with KL 1 or 2, and patients with KL 3 or 4, formed G0, G1, and G2 groups for KL classification, respectively. The selected features of the logistic LASSO regression at λ = λ_1se_ were compared between three groups of participants for each KOOS subscale in addition to KL classification.

### 2.6. Statistical Analysis

Descriptive statistics were used to describe the participants’ characteristics. The numerical variables (age, BMI, pain score, and KOOS) were presented as mean and standard deviation, and categorical variables (sex, KL classification, and the affected side) were shown as counts. The frequency domain features (ω and the power of the frequencies) were described as mean and range. Since the Shapiro–Wilk normality test did not confirm normality, univariate statistical comparisons were conducted using the non-parametric Wilcoxon rank sum test. In addition, the sex between the two groups was compared with a chi-square test. The mean and 95% confidence intervals (CI) were calculated for the differences in the means of the continuous variables (age, BMI, KOOS, and the frequency domain features of the signals). The significance level was considered as α = 0.05. Statistical analyses were conducted in the R Statistical Software.

## 3. Results

### 3.1. Participants

Table 2 compares the basic characteristics of the patient and control groups. The patients were significantly older and had higher BMI than the control group. In addition, the knee outcome scores were markedly higher in the control group compared with the patient group.

### 3.2. Frequency Content Comparison

Table 3 demonstrates the frequency domain features of the acceleration signals in the patients and controls. Except for the power of the first and fourth frequencies in the ML axis, the differences in the mean values for the calculated features were significant.

### 3.3. Frequency Content Feature Selection

**Figure 3:**
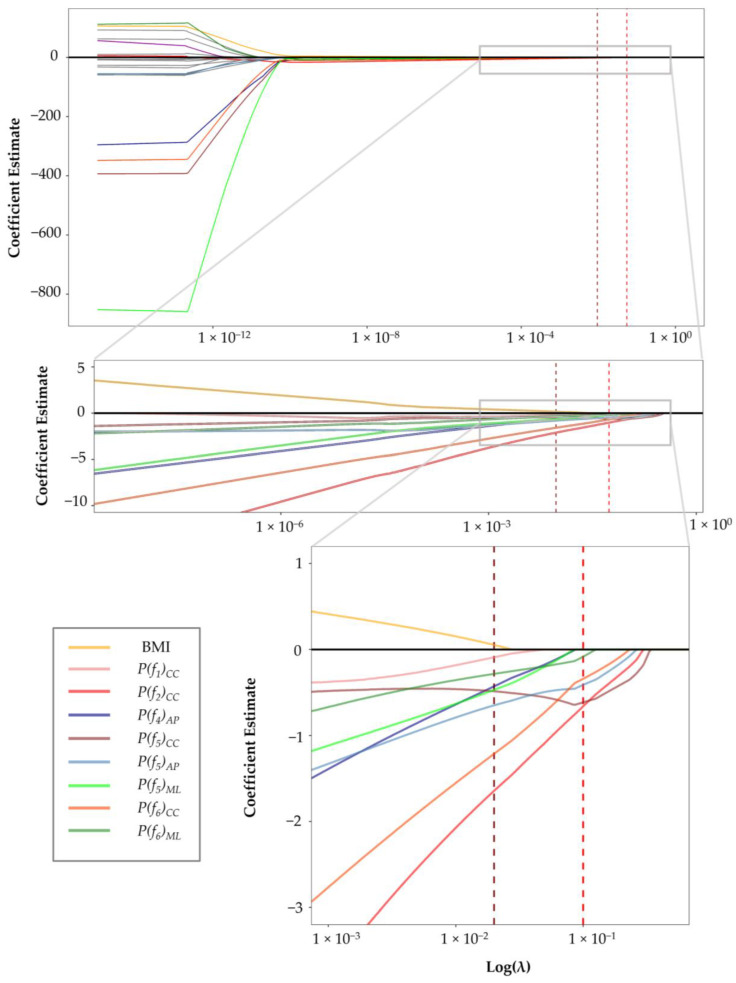
LASSO regression coefficient profile of the variables over different values of the penalty parameter (λ). Two vertical dashed lines demonstrate the values corresponding to λ_min_ (dark red), defined as the penalty value with minimum binomial deviance, and λ_1se_ (light red), defined as the largest penalty parameter within one standard error of the minimum binomial deviance.

Figure 3 shows the shrinkage in the estimate of the coefficients for different values of the penalty parameter (λ) in the logistic LASSO regression model. We could demonstrate that 13 out of 22 coefficients (including age, sex, ω, and certain signal frequency powers) vanished with λ less than 10^−8^. Increasing λ beyond λ_min_ led to excluding four other coefficients, among others, BMI. At λ_1se_, five variables remained in the model, out of which the power of the sixth frequency in the ML axis was close to zero and nullified soon afterward. Overall, four coefficients lasted in the model longest, i.e., the most determining features in distinguishing between the gait acceleration signals of osteoarthritic and non-osteoarthritis knees. These four features included the power of the second, fifth, and sixth frequencies of the CC axis and the power of the fifth frequency in the AP axis. The 95% CI and *p*-values for the coefficients at λ1se demonstrated similar results (Table 4).

### 3.4. Classification of Gait Accelerations

**Figure 4:**
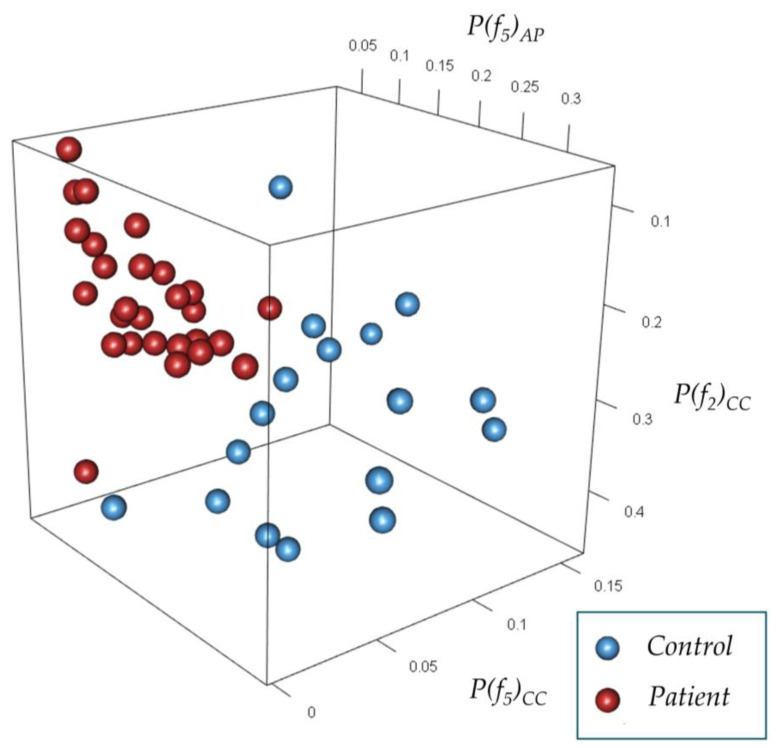
The cluster of the patients and controls based on the three most determining coefficients of the model: the power of the second and fifth frequency in the CC axis and the power of the fifth frequency in the AP axis.

The mean [95% CI] for the accuracy and Kappa of the logistic LASSO regression in classifying the gait acceleration signals into participants with and without knee OA were 0.91 [0.90, 0.92] and 0.72 [0.69, 074], respectively. The mean [95% CI] for the accuracy and Kappa of the model, containing only age, BMI, and ω as features, was 0.73 [0.72, 0.75] and 0.30 [0.27, 032], respectively. Figure 4 illustrates the distribution of the patients and controls based on the three most determining coefficients in the final model.

### 3.5. Selected Features vs. Severity of Knee OA

**Figure 5:**
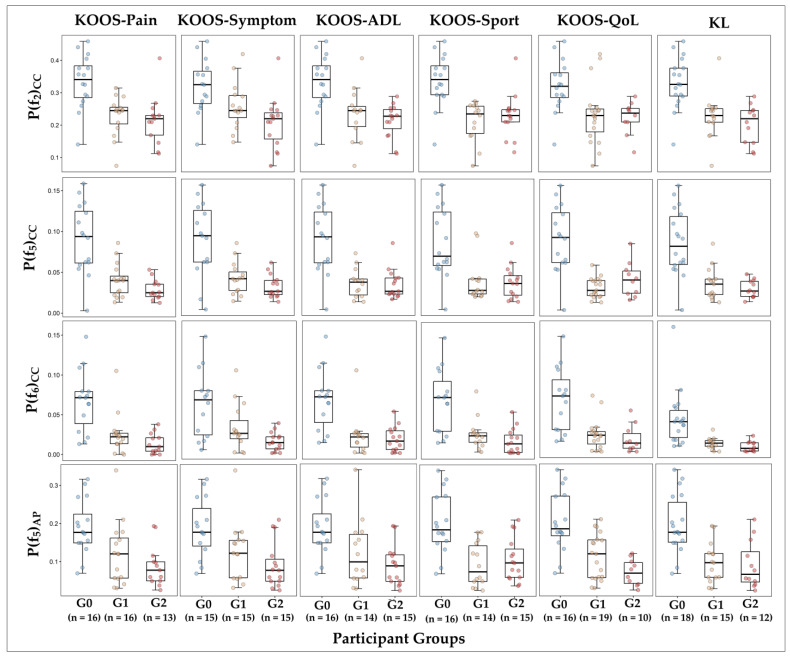
The distribution of the selected features in the final LASSO model between different groups of participants. G0: participants with mild or no knee OA, G1: participants with moderate knee OA, G2: participants with severe knee OA.

Figure 5 demonstrates the distribution of the selected features of the logistic LASSO regression model in three groups of participants with different severity of knee OA based on KOOS and KL classification (G0, G1, and G2). The features differed significantly between G0 and G1 groups and G0 and G2 groups divided by KOOS subscales and KL classification. However, in comparison between G1 and G2 groups (participants with moderate and severe knee OA), only the power of the sixth frequencies of the CC axis was statistically different between groups demarcated by KOOS-Symptoms.

## 4. Discussion

This study aimed to determine the ability of simple and low-sampling frequency IMUs to differentiate between individuals with and without knee OA. Using the signals’ frequency contents, we could distinguish between these individuals with high accuracy (0.91 ± 0.01). In addition, we could demonstrate differences in the distribution of the frequency domain features between individuals with different severity of knee OA.

We found the most significant differences in the acceleration frequency contents in the CC axis. The significance of the CC axis acceleration can be justified biomechanically by its direct relationship with stance phase knee joint compression forces, to which patients with knee OA or pre-OA are likely to be sensitive. In another study, Hung et al. observed higher tibial vertical acceleration in medial knee OA patients than age-matched controls, suggesting a more considerable ground impact on the knee joint [34]. They also observed significantly higher vertical acceleration differences between the tibia and femur in the patients compared to the control group, indicating a more significant kinetic moment between the segments, parallel to the clinical observation of the lateral thrust gait [34]. However, lower trunk-foot acceleration attenuation along the CC axis was observed in another study on an elderly female population without knee OA [35]. Levinger et al. also explored the frequency content of tibia acceleration signals [36]. They reported greater components in higher frequencies (>5 Hz) of the CC axis for the knee OA subjects than the healthy group. They attributed this finding to instability and altered attenuation of the impact during walking in patients with knee OA [36].

We also found different values for the selected features between patients with different severity of knee OA. However, the differences between the patients with moderate and severe knee OA were insignificant. We should emphasize that the KOOS performs best in measuring the changes in outcomes of the patients over time rather comparing different subjects [21]. Radiographic assessment is also an imprecise marker of pain or disability due to knee OA [37]. Nevertheless, inspecting the distribution of the selected features demonstrated that the values in the G1 group (moderate knee OA) were lower than the values in G0 (no/mild knee OA) and higher than in G2 (severe knee OA) groups divided by KOOS-Pain and KOOS-symptom. This pattern was also observed to a less degree in groups divided by KOOS-ADL and KL classification. Pain and symptom subscales, based on the International Classification of Functioning, Disability, and Health (ICF) framework, represent the body function (the anatomical and physiological level) [38]. While ADL shows activity (the personal level), and the sport and recreational and QoL subscales demonstrate participation (the level to which the person interacts with society) [38]. Different distribution of the selected features in patients with different knee OA severity creates the prospect of creating a gait deviation score based on frequency-domain features in these patients. Other studies evaluating the discriminating capacity of IMU data in knee OA severity assessed the time domain of the data [9,10] or employed more computationally complex approaches to extract the spatiotemporal parameters [6].

In the gait analysis application of machine learning, several methods have been described for extracting and selecting the features [39]. In this study, we employed logistic LASSO regression for feature selection and discriminating between the subjects with and without knee OA. As a logistic regression, this method can be used as a classification algorithm to distinguish between individuals with and without knee OA. LASSO can also perform feature selection by shrinking the features with little correlation with the response variable (i.e., the presence of knee OA in our study) toward zero. Logistic LASSO regression can handle the within-data correlation in gait data. Furthermore, logistic regression allows adjusting for the confounders [40]. Confounding variables, such as sex, age, and BMI, can affect gait characteristics [41,42,43], and researchers have applied different methods to control for the confounders in gait analysis [44]. We did not match the case and control groups for the confounding variables in this study. However, employing the LASSO regression, we could demonstrate that the confounders (sex, age, and BMI) were less effective than the frequency powers in the signal, and the effect of BMI (as the most influential confounder) was nullified before several frequency powers. Walking speed can also challenge gait analysis in knee OA [45]. In this study, we did not directly measure the gait velocity; however, ω, which correlates with the walking speed, was significantly higher in the controls compared with the patients. Nonetheless, the coefficient corresponding to ω was considerably lower than most of the frequency powers in the signal. The effect of ω was omitted by employing penalty values (λ) much smaller than the value we used in the final model in LASSO regression. Additionally, the inferior performance of a LASSO relying only on potential confounders (age, BMI, and ω), compared to the full LASSO model, signified the importance of the power of the frequencies in classifying the gait acceleration signals.

To our knowledge, this was the first study evaluating the frequency domain of IMU data to assess the presence and severity of knee OA and the first study employing a shrinkage technique for feature selection in gait analysis. Using LASSO regression, we identified the association between the frequency contents of the lower limb linear accelerations and ipsilateral knee OA. However, this study undeniably has several limitations. Firstly, conventional gait analysis was not performed as a ground truth to distinguish between the patients and controls. Nonetheless, the KOOS demonstrated significantly lower values, indicating walking problems in the patients. The correlation between KOOS and the severity of knee OA has been demonstrated [46]. Likewise, we could not compare the patients and the control group based on the radiographic features of the knee OA since the radiographic evaluation, for ethical reasons, was not performed in the control group. Therefore, the diagnosis of knee OA was overruled based on the absence of signs and symptoms [47]. An important limitation of not only this study but the frequency analysis is its non-intuitive nature compared to time-continuous analysis, complicating the interpretation of the frequency domain features. Relatedly, we must acknowledge that, based on the Nyquist theorem [48], the low-sampling frequency sensors provide us with limited bandwidth for data analysis. However, there is a trade between the sensor’s sampling rate and the length of available frequency bandwidth for analysis. Still, we believe finding differences in a limited bandwidth is impressive. Finally, we recorded gait data using standardized protocols in the hospital rather than at patients’ homes; however, we attempted to simplify the data collection protocol as much as possible to be reproducible in real-life environments.

Long-term monitoring of patients requires simple devices with long battery life. The IMU we utilized in this study was a single low-sampling accelerometer affixed by band-aid-like skin adhesion supplemented with a cloud connection for data transfer. The low-sampling accelerometers, as passive electronic components, have a long battery life of up to three months, which makes these devices an appropriate choice for the remote monitoring of patients. Despite the limitations, the Fourier coefficients of signals recorded by such sensors demonstrated a high discriminative capacity in knee OA. The method suggested in this study facilitates the automatic extraction of valuable parameters from IMU’s raw data for clinicians to use gait analysis in regular consultation. The IMU-derived parameters can help identify patients with knee OA and evaluate the response to treatments and interventions. In addition, these locomotion parameters provide an opportunity to automatically monitor the recovery process after surgery and intelligently individualize the rehabilitation programs beyond the limits of the clinics in real-life conditions.

However, before bringing this approach into patients’ homes, the correlation between the changes in the frequency contents of acceleration signals and the disease’s severity must be investigated. Before applying this method to remote monitoring of the patients, the responsiveness to changes in the signals’ frequency contents needs to be evaluated. The study outlines this vision that IMUs may provide practical information on the diagnosis and assessment of knee OA, consequently reducing the number of referrals to secondary healthcare and decreasing the cost and burden of diagnostic procedures. For instance, considering the location of the used sensors close to pants pockets, where most people carry their mobile phones, future studies can demonstrate the possibility of using smartphones equipped with inertial sensors instead of additional devices for diagnostic and investigative purposes in patients with knee OA.

## 5. Conclusions

The frequency contents of the lower limbs’ linear accelerations derived from the Fourier series of the signals can accurately differentiate between individuals with and without knee OA. However, despite the distribution of the frequency powers in patients with different severity of knee OA, further studies are required to clarify the correlation between the frequencies of IMU signals and the severity of knee OA.

## Figures and Tables

**Table 1:** The description of explanatory and outcome variables included in the logistic LASSO regression.

Variable		Type	Description
Explanatory variables	Age	Continuous	Age in years
	Sex	Binomial	Male/female
	BMI	Continuous	Body mass index as kg/m^2^
	ω	Continuous	The fundamental angular stride frequency of gait acceleration signals
	Power	Continuous	18 variables regarding the power of the signal at the first six frequencies of the CC ^1^, AP ^2^, and ML ^3^ axes
Outcome variable	Knee OA	Binomial	Yes/no

^1^ Craniocaudal; ^2^ Anteroposterior; ^3^ Mediolateral.

**Table 2:** Basic characteristics of the patients and controls.

Variable		Control (n = 18)	Patients (n = 27)	95% CI ^1^ (Differences of Means)	*p*-Value
**Female sex (n (%))**		11 (61)	15 (56)	-	0.9
Age (years)		60.8 ± 5.4	67.2 ± 9.4	(−10.9, −2.0)	0.006
BMI (kg/m^2^)		23.7 ± 3.0	27.7 ± 3.8	(−6.1 −2.0)	0.0003
Pain score ^2^	Ipsilateral knee	-	4.3 ± 2.2	-	-
	Contralateral knee	-	0.3 ± 0.7	-	-
KOOS ^3^	Pain	99.7 ± 0.9	53.6 ± 17.2	(36.1, 52.8)	<0.0001
	Symptom	96.8 ± 5.3	58.3 ± 23.2	(28.6, 46.4)	<0.0001
	ADL ^4^	99.3 ± 1.0	60.4 ± 16.4	(30.9, 48.5)	<0.0001
	Sport	96.7 ± 5.4	26.0 ± 20.5	(65.0, 80.0)	<0.0001
	QoL ^5^	97.2 ± 4.4	38.2 ± 15.3	(56.2, 62.5)	<0.0001
Knee OA severity ^6,7^	0 (n (%))	-	0	-	-
	1 (n (%))	-	7 (26)	-	-
	2 (n (%))	-	8 (30)	-	-
	3 (n (%))	-	9 (33)	-	-
	4 (n (%))	-	3 (11)	-	-
Painful knee	Right (n (%))	-	14 (52)	-	-
	Left (n (%))	-	13 (48)	-	-
Cadence (Steps/minute)		118 [112, 128]	109 [83, 134]	(5, 16)	0.001

^1^ 95% Confidence Interval; ^2^ Based on an 11-score numeric rating scale (0–10) in the patients. In the control group, we included participants without pain in the lower extremities, so we did not obtain pain scores in this group; ^3^ Knee injury and osteoarthritis outcome score; ^4^ Activities in daily living; ^5^ Quality of life; ^6^ The severity of knee osteoarthritis based on Kellgren–Lawrence classification; ^7^ We did not perform the radiographic evaluation in the control group. The presence of knee OA was ruled out in this group based on the absence of clinical signs and symptoms.

**Table 3:** The frequency domain features of the acceleration signals in the patients and controls and their 95% confidence intervals of differences of means.

Variable		Control (n = 18)	Patient (n = 27)	Differences of Means (95% CI)	*p*-Value
ω ^1^		6.16 [5.90, 6.68]	5.7 [4.34, 6.92]	(0.18, 0.64)	0.001
CC axis	*P*(*f*_1_)*_CC_*	0.15 [0.06, 0.25]	0.10 [0.02, 0.16]	(0.03, 0.07)	<0.0001
	*P*(*f*_2_)*_CC_*	0.33 [0.14, 0.46]	0.21 [0.07, 0.29]	(0.07, 0.16)	<0.0001
	*P*(*f*_3_)*_CC_*	0.20 [0.10, 0.30]	0.11 [0.01, 0.18]	(0.05, 0.12)	<0.0001
	*P*(*f*_4_)*_CC_*	0.11 [0.02, 0.46]	0.05 [0.01, 0.40]	(0.03, 0.07)	<0.0001
	*P*(*f*_5_)*_CC_*	0.08 [0.00, 0.14]	0.04 [0.01, 0.09]	(0.03, 0.06)	<0.0001
	*P*(*f*_6_)*_CC_*	0.08 [0.02, 0.30]	0.02 [0.00, 0.18]	(0.03, 0.07)	<0.0001
AP axis	*P*(*f*_1_)*_AP_*	0.16 [0.07, 0.35]	0.09 [0.02, 0.29]	(0.05, 0.10)	<0.0001
	*P*(*f*_2_)*_AP_*	0.42 [0.14, 0.58]	0.29 [0.05, 0.44]	(0.06, 0.18)	0.0002
	*P*(*f*_3_)*_AP_*	0.26 [0.07, 0.44]	0.19 [0.02, 0.33]	(0.01, 0.12)	0.02
	*P*(*f*_4_)*_AP_*	0.26 [0.11, 0.57]	0.12 [0.02, 0.44]	(0.06, 0.18)	0.0001
	*P*(*f*_5_)*_AP_*	0.20 [0.07, 0.34]	0.09 [0.01, 0.21]	(0.08, 0.15)	<0.0001
	*P*(*f*_6_)*_AP_*	0.10 [0.01, 0.36]	0.04 [0.00, 0.22]	(0.01, 0.09)	0.003
ML axis	*P*(*f*_1_)*_ML_*	0.08 [0.01, 0.15]	0.06 [0.00, 0.13]	(−0.01, 0.04)	0.2
	*P*(*f*_2_)*_ML_*	0.12 [0.02, 0.26]	0.07 [0.02, 0.17]	(0.03, 0.08)	0.003
	*P*(*f*_3_)*_ML_*	0.09 [0.03, 0.15]	0.04 [0.00, 0.09]	(0.03, 0.07)	0.0002
	*P*(*f*_4_)*_ML_*	0.07 [0.00, 0.20]	0.05 [0.00, 0.17]	(−0.01, 0.04)	0.3
	*P*(*f*_5_)*_ML_*	0.06 [0.02, 0.22]	0.02 [0.00, 0.07]	(0.01, 0.05)	0.005
	*P*(*f*_6_)*_ML_*	0.08 [0.00, 0.28]	0.03 [0.00, 0.15]	(0.03, 0.06)	0.0001

^1^ The fundamental angular stride frequency.

**Table 4:** The coefficients of the logistic LASSO regression at λ = λ _1se_.

Variable		Coefficient [95% CI]	*p*-Value
Sex		-	-
Age		-	-
BMI		-	-
ω		-	-
CC axis	*P*(*f*_1_)*_CC_*	-	-
	*P*(*f*_2_)*_CC_*	−0.66 [−0.81, −0.50]	<0.0001
	*P*(*f*_3_)*_CC_*	-	-
	*P*(*f*_4_)*_CC_*	-	-
	*P*(*f*_5_)*_CC_*	−0.62 [−0.96, −0.39]	<0.0001
	*P*(*f*_6_)*_CC_*	−0.34 [−0.46, −0.21]	<0.0001
AP axis	*P*(*f*_1_)*_AP_*	-	-
	*P*(*f*_2_)*_AP_*	-	-
	*P*(*f*_3_)*_AP_*	-	-
	*P*(*f*_4_)*_AP_*	-	-
	*P*(*f*_5_)*_AP_*	−0.41 [−0.62, −0.22]	<0.0001
	*P*(*f*_6_)*_AP_*	-	-
ML axis	*P*(*f*_1_)*_ML_*	-	-
	*P*(*f*_2_)*_ML_*	-	-
	*P*(*f*_3_)*_ML_*	-	-
	*P*(*f*_4_)*_ML_*	-	-
	*P*(*f*_5_)*_ML_*	-	-
	*P*(*f*_6_)*_ML_*	−0.08 [−0.13, 0.07]	0.1

## Data Availability

The data presented in this study are available upon request from the corresponding author.

## References

[1] Mills K., Hunt M.A., Ferber R. (2013). Biomechanical deviations during level walking associated with knee osteoarthritis: A systematic review and meta-analysis. Arthritis Care Res..

[2] Renggli D., Graf C., Tachatos N., Singh N., Meboldt M., Taylor W.R., Stieglitz L., Daners M.S. (2020). Wearable Inertial Measurement Units for Assessing Gait in Real-World Environments. Front. Physiol..

[3] Rose M.J., Costello K.E., Eigenbrot S., Torabian K., Kumar D. (2022). Inertial Measurement Units and Application for Remote Health Care in Hip and Knee Osteoarthritis: Narrative Review. JMIR Rehabil. Assist. Technol..

[4] Ismailidis P., Egloff C., Hegglin L., Pagenstert G., Kernen R., Eckardt A., Ilchmann T., Mündermann A., Nüesch C. (2020). Kinematic changes in patients with severe knee osteoarthritis are a result of reduced walking speed rather than disease severity. Gait Posture.

[5] Odonkor C., Kuwabara A., Tomkins-Lane C., Zhang W., Muaremi A., Leutheuser H., Sun R., Smuck M. (2020). Gait features for discriminating between mobility-limiting musculoskeletal disorders: Lumbar spinal stenosis and knee osteoarthritis. Gait Posture.

[6] Tadano S., Takeda R., Sasaki K., Fujisawa T., Tohyama H. (2016). Gait characterization for osteoarthritis patients using wearable gait sensors (H-Gait systems). J. Biomech..

[7] Van Der Straaten R., Wesseling M., Jonkers I., Vanwanseele B., Bruijnes A., Malcorps J., Bellemans J., Truijen J., De Baets L., Timmermans A. (2020). Functional movement assessment by means of inertial sensor technology to discriminate between movement behaviour of healthy controls and persons with knee osteoarthritis. J. Neuroeng. Rehabil..

[8] Tanimoto K., Takahashi M., Tokuda K., Sawada T., Anan M., Shinkoda K. (2017). Lower limb kinematics during the swing phase in patients with knee osteoarthritis measured using an inertial sensor. Gait Posture.

[9] Barrois R., Gregory T., Oudre L., Moreau T., Truong C., Pulini A.A., Vienne A., Labourdette C., Vayatis N., Buffat S. (2016). An automated recording method in clinical consultation to rate the limp in lower limb osteoarthritis. PLoS ONE.

[10] Na A., Buchanan T.S. (2021). Validating Wearable Sensors Using Self-Reported Instability among Patients with Knee Osteoarthritis. PM R.

[11] Honert E.C., Pataky T.C. (2021). Timing of gait events affects whole trajectory analyses: A statistical parametric mapping sensitivity analysis of lower limb biomechanics. J. Biomech..

[12] Pataky T.C. (2010). Generalized n-dimensional biomechanical field analysis using statistical parametric mapping. J. Biomech..

[13] Deluzio K.J., Astephen J.L. (2007). Biomechanical features of gait waveform data associated with knee osteoarthritis. An application of principal component analysis. Gait Posture.

[14] Begg R., Kamruzzaman J. (2005). A machine learning approach for automated recognition of movement patterns using basic, kinetic and kinematic gait data. J. Biomech..

[15] Agostini V., Nascimbeni A., Gaffuri A., Imazio P., Benedetti M.G., Knaflitz M. (2010). Normative EMG activation patterns of school-age children during gait. Gait Posture.

[16] Wong M.A., Simon S., Olshen R.A. (1983). Statistical analysis of gait patterns of persons with cerebral palsy. Stat. Med..

[17] Long J.T., Klein J.P., Sirota N.M., Wertsch J.J., Janisse D., Harris G.F. (2004). Biomechanics of the Double Rocker Sole Shoe: Gait Kinematics and Kinetics. Conf. Proc. IEEE Eng. Med. Biol. Soc..

[18] Giakas G., Baltzopoulos V. (1997). Time and frequency domain analysis of ground reaction forces during walking: An investigation of variability and symmetry. Gait Posture.

[19] Schneider E., Chao E.Y. (1983). Fourier analysis of ground reaction forces in normals and patients with knee joint disease. J. Biomech..

[20] Chao E., Bergmann G., Kölbel R., Rohlmann A. (1987). Gait analysis: A survey. Biomechanics: Basic and Applied Research, Selected Proceedings of the Fifth Meeting, European Society of Biomechanics, Berlin, Germany, 8–10 September 1986.

[21] Roos E.M., Roos H.P., Lohmander L.S., Ekdahl C., Beynnon B.D. (1998). Knee Injury and Osteoarthritis Outcome Score (KOOS)--development of a self-administered outcome measure. J. Orthop. Sports Phys. Ther..

[22] Kohn M.D., Sassoon A.A., Fernando N.D. (2016). Classifications in Brief: Kellgren-Lawrence Classification of Osteoarthritis. Clin. Orthop. Relat. Res..

[23] Ghaffari A., Rahbek O., Lauritsen R.E.K., Kappel A., Kold S., Rasmussen J. (2022). Criterion Validity of Linear Accelerations Measured with Low-Sampling-Frequency Accelerometers during Overground Walking in Elderly Patients with Knee Osteoarthritis. Sensors.

[24] Pedersen B.S., Kristensen M.T., Josefsen C.O., Lykkegaard K.L., Jønsson L.R., Pedersen M.M. (2022). Validation of Two Activity Monitors in Slow and Fast Walking Hospitalized Patients. Rehabil. Res. Pract..

[25] Winter D.A. (2009). Biomechanics and Motor Control of Human Movement.

[26] Derrick T.R., Robertson D.G.E., Caldwell G.E., Hamil J., Kamen G., Whittlesey S.N. (2013). Signal Processing. Research Methods in Biomechanics.

[27] Van Rossum G., Drake F.L. (2009). Python 3 Reference Manual.

[28] Tibshirani R. (1996). Regression Shrinkage and Selection via the Lasso. J. R. Stat. Soc. Ser. B.

[29] R Core Team (2022). R: A Language and Environment for Statistical Computing.

[30] Friedman J.H., Hastie T., Tibshirani R. (2010). Regularization Paths for Generalized Linear Models via Coordinate Descent. J. Stat. Softw..

[31] Kuhn M. (2008). Building Predictive Models in R Using the caret Package. J. Stat. Softw..

[32] Simon N., Friedman J., Hastie T., Tibshirani R. (2011). Regularization Paths for Cox’s Proportional Hazards Model via Coordinate Descent. J. Stat. Softw..

[33] Taylor J., Tibshirani R. (2016). Post-selection inference for L1-penalized likelihood models. Can. J. Stat..

[34] Hung S.W., Shih Y.F., Chiang W.H., Chen W.Y. (2015). Vertical and mediolateral knee acceleration during level walking in individuals with medialcompartment knee osteoarthritis and a lateral thrust gait. Physiotherapy.

[35] Zhang Y., Zhou X., Pijnappels M., Bruijn S.M. (2021). Differences in Gait Stability and Acceleration Characteristics between Healthy Young and Older Females. Front. Rehabil. Sci..

[36] Levinger P., Lai D.T.H., Begg R., Menz H., Feller J., Webster K. Fourier analysis of tibia acceleration in subjects with knee oste-oarthritis: Preliminary results. Proceedings of the ISSNIP 2008—2008 International Conference on Intelligent Sensors, Sensor Networks and Information Processing.

[37] Bedson J., Croft P.R. (2008). The discordance between clinical and radiographic knee osteoarthritis: A systematic search and summary of the literature. BMC Musculoskelet. Disord..

[38] World Health Organization (WHO) (2001). International Classification of Functioning, Disability, and Health.

[39] Khera P., Kumar N. (2020). Role of machine learning in gait analysis: A review. J. Med. Eng. Technol..

[40] Pourhoseingholi M.A., Baghestani A.R., Vahedi M. (2012). How to control confounding effects by statistical analysis. Gastroenterol. Hepatol. Bed Bench..

[41] Kobsar D., Barden J.M., Clermont C., Wilson J.L.A., Ferber R. (2022). Sex differences in the regularity and symmetry of gait in older adults with and without knee osteoarthritis. Gait Posture.

[42] Judge J.O., Ounpuu S., Davis R.B. (1996). Effects of Age on the Biomechanics and Physiology of Gait. Clin. Geriatr. Med..

[43] Rosso V., Agostini V., Takeda R., Tadano S., Gastaldi L. (2019). Influence of BMI on Gait Characteristics of Young Adults: 3D Evalu-ation Using Inertial Sensors. Sensors.

[44] Kobsar D., Charlton J.M., Hunt M.A. (2019). Individuals with knee osteoarthritis present increased gait pattern deviations as measured by a knee-specific gait deviation index. Gait Posture.

[45] Astephen Wilson J.L. (2012). Challenges in dealing with walking speed in knee osteoarthritis gait analyses. Clin. Biomech..

[46] Oishi K., Tsuda E., Yamamoto Y., Maeda S., Sasaki E., Chiba D., Takahashi I., Nakaji S., Ishibashi Y. (2016). The Knee injury and Osteoarthritis Outcome Score reflects the severity of knee osteoarthritis better than the revised Knee Society Score in a general Japanese population. Knee.

[47] Zhang W., Doherty M., Peat G., Bierma-Zeinstra M.A., Arden N.K., Bresnihan B., Herrero-Beaumont G., Kirschner S., Leeb B.F., Lohmander S. (2010). EULAR evidence-based recommendations for the diagnosis of knee osteoarthritis. Ann. Rheum. Dis..

[48] Nyquist H., Nyquist H. (1928). Certain Topics in Telegraph Transmission Theory. Trans. Am. Inst. Electr. Eng..

